# Toward a Genetic Signature of Resistance to Activity‐Based Anorexia in Striatal Projecting Cortical Neurons

**DOI:** 10.1002/eat.24538

**Published:** 2025-09-05

**Authors:** K. Huang, M. A. Magateshvaren Saras, K. Conn, E. Greaves, F. Reed, S. Tyagi, H. Munguba, C. J. Foldi

**Affiliations:** ^1^ Department of Physiology Monash University Clayton Victoria Australia; ^2^ Monash Biomedicine Discovery Institute Clayton Victoria Australia; ^3^ IITB‐Monash Research Academy Mumbai Maharashtra India; ^4^ Monash University, Central Clinical School Melbourne Victoria Australia; ^5^ Australian Eating Disorders Research and Translation Centre University of Sydney Sydney New South Wales Australia; ^6^ RMIT School of Computing Technologies Melbourne Victoria Australia; ^7^ Department of Neuroscience, Physiology & Pharmacology University College London London UK

**Keywords:** activity‐based anorexia, anorexia nervosa, cortico‐striatal circuits, disease models (animal), gene transcription, signaling pathways

## Abstract

**Objective:**

Converging evidence from neuroimaging studies and genome‐wide association study (GWAS) suggests the involvement of prefrontal cortex (PFC) and striatum dysfunction in the pathophysiology of anorexia nervosa (AN). However, identifying the causal role of circuit‐specific genes in the development of the AN‐like phenotype remains challenging and requires the combination of novel molecular tools and preclinical models.

**Methods:**

We used the activity‐based anorexia (ABA) rat model in combination with a novel viral‐based translating ribosome affinity purification (TRAP) technique to identify transcriptional differences within a specific neural pathway that we have previously demonstrated to mediate pathological weight loss in ABA rats (i.e., medial PFC neurons that project to the nucleus accumbens shell). We compared actively transcribed genes in rats susceptible to weight loss to the subpopulation of rats resistant to weight loss under the same experimental conditions.

**Results:**

We reveal 1424 differentially expressed genes between Susceptible and Resistant rats, highlighting important transcriptional changes associated with ABA within this pathway. The changes observed were independent of current calorie deficit and associated with metabolic, mitochondrial, and neural functions. Further, we show that genes upregulated in Resistant rats were involved in mitochondrial function, while downregulated genes were associated with cytoskeletal, postsynaptic, and axonal functions, supporting the hypothesis that hyperexcitability of cortico‐striatal circuit function is a critical mediator of pathological weight loss in ABA.

**Discussion:**

These findings represent an essential first step in understanding how circuit‐specific gene expression patterns may contribute to susceptibility to ABA and provide potential molecular targets for manipulation in this animal model of AN.

**Public Significance:**

This study identifies specific brain gene activity patterns that may explain why some individuals are more vulnerable to extreme weight loss, as seen in AN. Using an advanced molecular technique in a well‐established animal model, key differences in a neural pathway linked to cognitive control were observed. These findings pave the way for more targeted treatments that could prevent or reverse this dangerous condition.

**Summary**

- Transcriptional differences within medial prefrontal cortex to nucleus accumbens shell (mPFC–AcbSh) neurons distinguish rats highly susceptible to activity‐based anorexia (ABA) from those resistant to pathological weight loss.
- Resistant rats showed upregulation of mitochondrial function genes, while susceptible rats had upregulation of genes related to synaptic structure and signaling, implicating excitability of this circuit in driving maladaptive weight loss behavior.
- Transcriptomic changes align with human genome‐wide association study (GWAS) findings and support links between anorexia nervosa and both metabolic and psychiatric comorbidities.
- The study highlights potential molecular targets for future gene manipulation and therapeutic intervention. It also provides a foundation for creating refined genetic animal models that integrate multiple AN‐associated variants to better reflect the polygenic nature of the disorder.

## 1 Introduction

Anorexia nervosa (AN) is a complex and serious illness characterized by a low body‐mass index, behavioral disturbances such as inflexible thinking and compulsive behaviors, and higher mortality rates than most psychiatric disorders (Chesney et al. 2014). Available treatments have low rates of clinical success, in part, because renourishment protocols are not efficacious in addressing the deleterious drive to exacerbate dangerously low body weights (Watson and Bulik 2013). Converging evidence from human neuroimaging studies and preclinical models suggests the involvement of altered neural circuit function, particularly aberrant activity within the prefrontal cortex (PFC) and striatum regions of the brain (Cha et al. 2016; Ehrlich et al. 2015; Foldi et al. 2017; Milton et al. 2021). Interestingly, the largest genome‐wide association study (GWAS) conducted to date (including ~17K cases and 55K controls) (Watson et al. 2019) suggests that genes associated with AN are enriched in similar brain regions, namely PFC and striatum (Song et al. 2021), activity in which is associated with food reward valuation and cognitive flexibility (Cox and Witten 2019; Joshi et al. 2022). Although GWAS findings are informative, identifying strong hypotheses about their connections to specific genes, or how these specific genes are causal to changes in brain function or behavior is not straightforward. Using a sophisticated complement of analytical tools including expression quantitative trait loci (eQTL) and chromosome conformation capture (Hi‐C) interactions, 58 specific genes were identified to be significantly dysregulated in AN cases, with the clearest evidence for a role in the etiology of AN for *Cadm1*, *Mgmt*, *Focp1*, and *Ptbp2* (Watson et al. 2019), genes that are involved in cellular processes like receptor binding, DNA repair, and cell‐type specific gene transcription during development. However, it is important to recognize that by definition these findings are associations and may reflect variants and genes with no direct biological relevance to the causes of AN. Moreover, understanding the genetic predisposition of any neuropsychiatric disease is insufficient for informing novel prevention or treatment strategies if it does not pinpoint the brain regions or circuits where those genes have the greatest impact. Insight into the causal role of circuit‐specific genes in the development and/or maintenance of AN‐like phenotypes is now possible with novel molecular tools in combination with preclinical models.

Activity‐based anorexia (ABA) is a biobehavioral rodent model that recapitulates many of the behavioral phenotypes of AN in humans, including voluntary food restriction and paradoxical hyperactivity in states of negative energy balance that lead to rapid reductions in body weight. Accordingly, the ABA model has been used for decades to gain mechanistic insight into the biological bases for AN, including predisposing factors (Barbarich‐Marsteller et al. 2013; Carrera et al. 2006; Hancock and Grant 2009; Milton et al. 2022). The observation that when exposed to the same environmental conditions of ABA, both wild‐type adolescent rats (Milton et al. 2018) and mice (Beeler and Burghardt 2021) consistently split into ‘Susceptible’ and ‘Resistant’ phenotypes makes this a particularly powerful model for understanding what differentiates individuals that go on to develop pathological weight loss and compulsive exercise from those that do not. Therefore, the ABA model could provide a valuable tool to dissect the causal involvement of genes associated with AN, but thus far its use has not shed sufficient light on the genetic determinants of AN (Foldi 2024). We have previously shown that suppressing activity within a cortico‐striatal neural circuit that extends between the medial prefrontal cortex (mPFC) and nucleus accumbens shell (AcbSh) both prevents weight loss in ABA and improves cognitive flexibility in female rats (Milton et al. 2021). This circuit is important in the context of AN‐like behaviors because of its role in the suppression of learned stimulus–response behavior (i.e., behavior that is influenced by the consequences of actions) by regulating sensitivity to punishment (Piantadosi et al. 2020). Moreover, dysfunction in this circuit is reflected in the analogous human brain regions (PFC, ventral striatum) in individuals with AN, which may increase throughout the course of illness (Fladung et al. 2013) and persist after weight recovery (Ehrlich et al. 2015). A caveat in interpreting our circuit‐level findings arises from a subsequent study in which we examined flexible learning and susceptibility to ABA in the same (rat) subjects. Here, we showed that in fact *better* performance on the cognitive task predicted weight loss in ABA (Huang et al. 2023) suggesting a more nuanced role for cortico‐striatal circuitry and reinforcement learning in the development of ABA and possibly the prodromal symptoms of AN in humans.

In order to interpret how disrupted neurochemistry and/or neural circuit function gives rise to the specific pathology of AN, the molecular phenotype that determines the functional output of the neural circuits involved needs to be identified. Traditional assessment of the genetic contributions to AN using targeted genetic knockout/down in animal models has yielded insufficient evidence to date for a molecular signature of AN (Scharner and Stengel 2021), perhaps because of compensatory mechanisms that come into play when a gene construct is manipulated within an entire organism or brain region. It is also important in the context of a condition like AN, for which very little is known about the underlying genetic drivers, to perform an unbiased screen of gene expression within specific circuits of ABA rats, in order to reveal novel mechanisms. With this in mind, we utilized translating ribosome affinity purification (TRAP) technology, which allows the synthesis of molecular and neuroanatomical information by immunoprecipitation of translating mRNAs from any population of neurons that express enhanced green fluorescent protein (eGFP). While this technique has been traditionally used to identify cell type‐specific genetic profiles (Heiman et al. 2014) more recently it has been used effectively to differentiate genetic signatures of addiction‐like behavior to non‐addicted controls (Kawa et al. 2025). Thus, in order to identify differentially regulated genes *within a specific neural pathway* that modulates weight loss in ABA rats, we employed a dual viral approach, in which coincident injection of a retrogradely transporting Cre‐expressing virus and a Cre‐dependent TRAP construct (eGFP‐tagged ribosome protein L10a; EGFPL10a) were injected into the AcbSh and mPFC, respectively. This allows eGFP‐tagging of ribosomes, and in turn access to actively translating mRNA, specifically within neurons of the mPFC‐AcbSh pathway. RNA sequencing of the immunoprecipitated polysomes was performed to identify a circuit‐based genetic profile that is associated with Susceptibility or Resistance to ABA. This type of approach represents a critical first step in the pursuit of understanding the *causes* of functional changes in reward and cognitive processes involved in pathological weight loss. Our findings pave the way for validation and manipulation of specific genes in the ABA model in future studies to identify causal molecular mechanisms.

## 2 Materials and Methods

### 2.1 Animals and Housing

**FIGURE 1:**
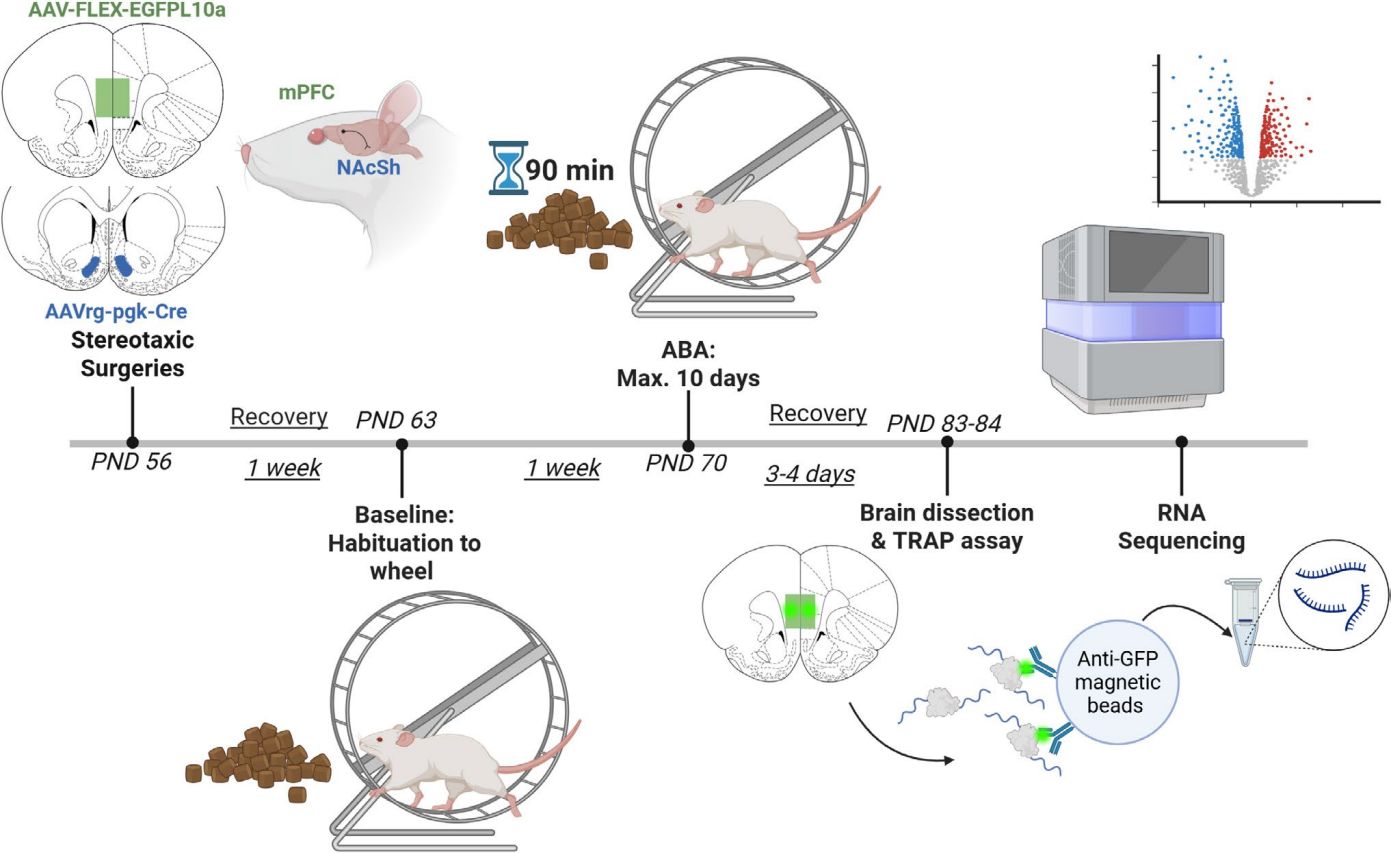
Timeline of experiments. Female Sprague–Dawley rats were injected with AAV‐FLEX‐EGFPL10a into medial prefrontal cortex (mPFC) and retrogradely transporting AAVrg‐pgk‐Cre into nucleus accumbens shell (NAcSh). Following 1 week of recovery, rats were habituated to running wheels at PND 63 with ad libitum food access for 7 days to determine baseline body weight and running wheel activity. This age corresponds to late adolescence to young adulthood in a human (approximately 18 years). Activity‐based anorexia (ABA) conditions commenced at PND 70 (approximately 20 human years) with food access limited to 90 min per day and lasted for a maximum of 10 days or until rats reached < 80% baseline body weight. Rats were allowed to recover to baseline body weight with ad libitum food access for 3–4 days before brain dissection at PND 83–84; approximately 24 human years). Tissue from mPFC was used in the TRAP assay to extract labeled RNA, followed by RNA sequencing and analyses.

All animals were obtained from the Monash Animal Research Platform (MARP; Clayton, VIC, Australia). Female Sprague–Dawley rats (*n* = 28) were 7 weeks of age (postnatal day 49; PND 49) on arrival in the laboratory. Animals were group‐housed and acclimated to the 12‐h light/dark cycle (lights off at 1100 h) for 7 days in a temperature (22°C–24°C) and humidity (30%–50%) controlled room before experiments commenced at 8 weeks of age (PND 56). Rats reach sexual maturity at PND 50; therefore, the experimental age of 8 weeks corresponds to approximately 16–20 years old in a human (Agoston 2017). Functionally, the age range of rats used in these experiments captures the post‐pubertal (but not fully mature) phase seen in human adolescence and represents a common period of emergence of AN in humans (Grilo and Udo 2021). A male rat was individually housed in all experimental rooms to facilitate synchronization of the oestrous cycle of the female rats (Cora et al. 2015), and although cycle stage was not directly assessed in the present study, the within‐ABA comparison design controls for the known disruption that ABA exposure has on regular cycling (Dixon et al. 2003). All experimental procedures (see Figure 1 for timeline) were conducted in accordance with the Australian Code for the care and use of animals for scientific purposes and approved by the Monash Animal Resource Platform Ethics Committee (ERM 29143).

### 2.2 Stereotaxic Surgeries

**FIGURE 2:**
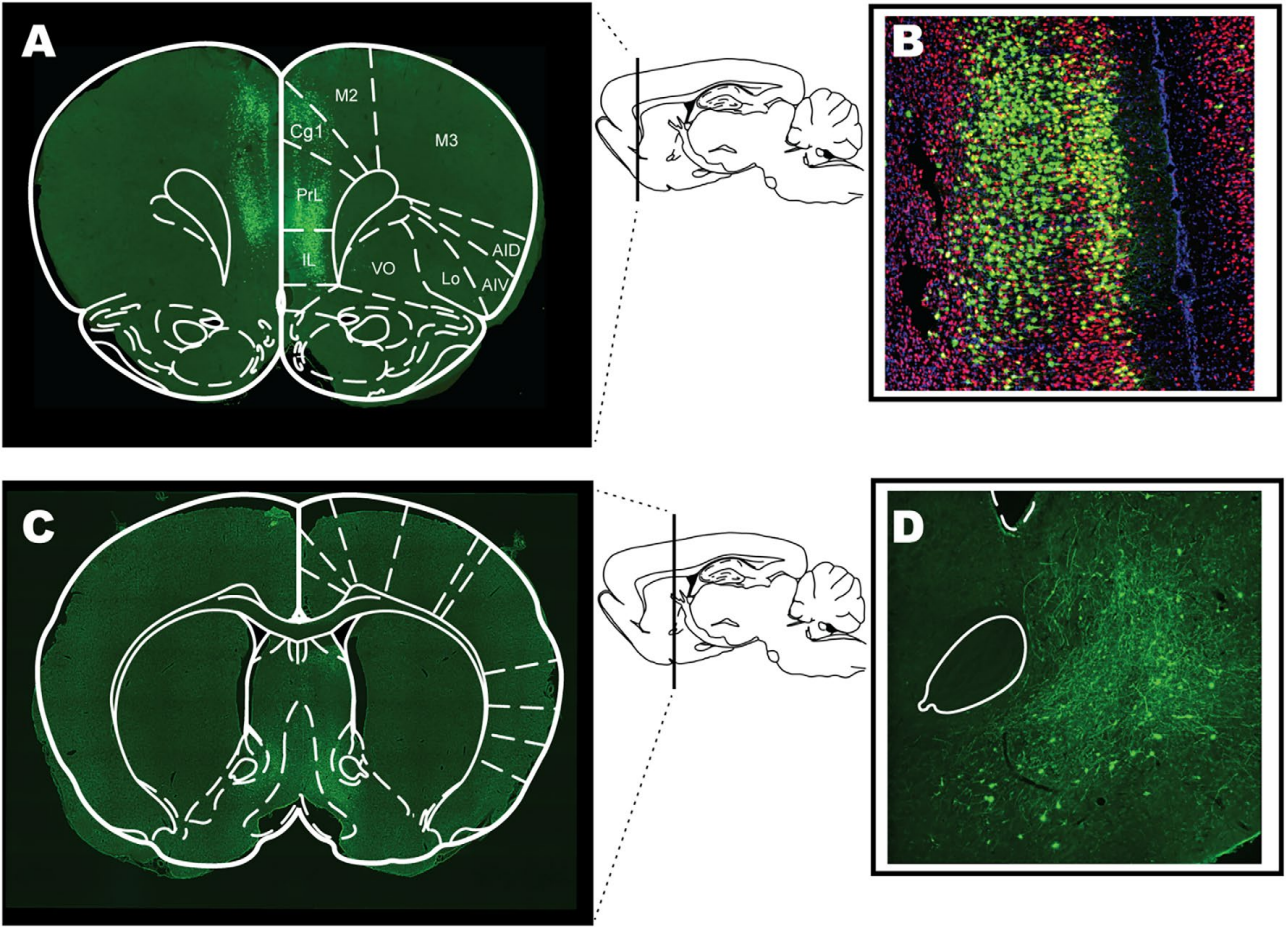
EGFPL10a (TRAP) expression is restricted to the medial prefrontal cortex (mPFC). (A) The mPFC region labeled with EFGP collected for RNA sequencing with atlas overlay (Bregma 3.20 mm). (B) Micrograph (20×) of the mPFC midline with eGFPL10a (green), NeuN (red) and DAPI (blue) immunofluorescence. (C) The medial AcbSh region confirming terminal staining in the mPFC‐AcbSh pathway with atlas overlay (Bregma 0.48 mm). (D) Micrograph (20×) of terminal EGFP staining in the AcbSh [markup to orient to the anterior commissure bundle (solid line) and lateral ventricle (dashed line)].

Stereotaxic surgeries were performed under anesthesia (2.5%–3% isoflurane in oxygen) using a stereotaxic apparatus (Kopf Instruments, CA, USA) and micropipettes pulled from borosilicate glass (0.64 mm; Drummond Scientific, PA, USA) with a tip of ~40 μm. All animals were injected bilaterally with 250 nL of retro‐Cre (AAVrg‐pgk‐Cre; Addgene, #24593) into AcbSh (from bregma and brain surface: anteroposterior: +1.6 mm, mediolateral: ±0.7 mm, dorsoventral: −6.6 mm), followed by bilateral injection of 250 nL of the Cre‐dependent TRAP construct (AAV5‐FLEX‐EGFPL10a; Addgene #98747) into the mPFC (from bregma and brain surface: anteroposterior: +2.8 mm, mediolateral: ±0.5 mm, dorsoventral: −3.2 mm). Viral constructs were infused over 5 min (50 nL/min), with pipette tips left at the coordinates for a further 5 min to allow virus dispersal. Meloxicam (2 mg/kg; Boehringer Ingelheim, Germany) was subcutaneously injected into animals during surgery. Suture clips were used to close the surgery sites. Animals were group‐housed with ad libitum food and water access post‐surgery for 7 days to recover and allow for viral expression (see Figure 2 for validation of expression and Supporting Information: Supplementary Methods).

### 2.3 Activity‐Based Anorexia (ABA)

Rats were individually housed in transparent living chambers with a removable food basket and a running wheel (Lafayette Instruments, IN, USA). Rats were allowed to habituate to the living chamber with ad libitum food access and a locked running wheel for 1 day, followed by habituation to the unlocked running wheel for 7 days to determine baseline activity. Running wheel activity was recorded in revolutions by the Scurry Activity Wheel Software (Lafayette Instruments, IN, USA) with data extracted in 1 h time bins. During ABA, food access was restricted to 90 min per day at the onset of the dark phase (1100–1230 h), with body weight measurements taken daily at 1100 h. Running in the hour before the feeding window (1000–1100 h) was considered food anticipatory activity (FAA) (Milton et al. 2021). Time‐restricted food access persisted for a maximum of 10 days or until animals reached < 80% of baseline body weight (ABA criterion), the baseline measure being that taken on Day 0 (prior to the first food restriction period). All rats (including those resistant to developing ABA) were refed with ad libitum food access allowing them to regain body weight to at least 100% of baseline (minimum of 3 days) and running wheel access. Following this, rats were euthanized with 300 mg/kg sodium pentobarbitone (Lethabarb; Virbac, Australia), followed by rapid dissection of the mPFC region performed in dissection buffer containing 1× HBSS, 2.5 mM HEPES‐KOH (pH = 7.4), 35 mM glucose, 4 mM NaHCO_3_ and 100 μg/mL cycloheximide (CHX). Dissected tissue was snap frozen in liquid nitrogen and stored at −80°C until use.

### 2.4 Translating Ribosome Affinity Purification (TRAP) Assay and RNA Sequencing

TRAP was performed based on previously described protocols (Heiman et al. 2014; Ip et al. 2019; Nectow et al. 2017), followed by RNA extraction using the RNeasy Micro Kit (Qiagen, The Netherlands). Twelve samples were strategically selected for sequencing according to weight loss profiles, in which equal numbers (*n* = 4/group) were either clearly Resistant to developing ABA, rapidly lost weight under conditions of ABA, and were removed from the experimental paradigm after 3–4 days (Susceptible) or exhibited an Intermediate phenotype, in which they lost weight to reach the removal criterion but more slowly than Susceptible rats (6–9 days). Rats with ambiguous weight loss profiles were excluded from the sequencing cohort to enable consistent group sizes with the Resistant rats that typically constitute ~30% of ABA cohorts (Milton et al. 2018) (Hurley et al. 2022). Total RNA samples underwent quality control by bioanalyzer, and the resulting library pool was QC'd by Qubit, Bioanalyzer, and qPCR. Libraries were made by individual first strand synthesis to add the i7 index sequences indicated in Table S1.

### 2.5 Analysis

The raw sequencing reads were adapter trimmed using Trim Galore (v0.6.7; https://github.com/FelixKrueger/TrimGalore) with standard parameters. Reads were aligned to the Rat reference genome (Rattus_norvegicus.mRatBN7) with STAR (v 2.7.9a) (Dobin et al. 2013) and quantified using Salmon (v 1.9.0) (Patro et al. 2017). The reference genome was downloaded from Ensembl, along with the gene annotation in a .gtf file. MultiQC (v 3.9.5) (Ewels et al. 2016) was used to generate the summary of the final quality metrics for all datasets. Differential expression analysis was carried out using edgeR (Robinson et al. 2010) along with RUVseq (Risso et al. 2014) to remove unwanted noise in the data. Genes with one count per million in at least three samples per group were selected to perform the tests of differential expression. Significant differentially expressed genes (DEGs) were selected using log‐fold‐change of ±1 and FDR cut‐off of 0.05 and were used for performing gene‐set enrichment analysis (GSEA). The Rat Genome Database pathway and gene ontology (GO) analysis utilities were used (Petri et al. 2011). GSEA was used to identify classes of genes that are over‐represented in a large set of genes or proteins and may have an association with different phenotypes. GO analyses were summarized at the level of Biological Process (BP), cellular component (CC), and molecular function (MF). Pathway analysis results were plotted using the online platform SRplot (http://www.bioinformatics.com.cn/SRplot) (Tang et al. 2023). Specific details of quality metrics from the MultiQC report are available on request.

### 2.6 Statistical Analysis

Statistical analyses were performed using GraphPad Prism (GraphPad Software, San Diego, CA, USA). Statistical significance was considered as *p* < 0.05. Analyses including two‐tailed unpaired *t*‐test, one‐way and two‐way analysis of variance (ANOVA) with Tukey's or Bonferroni's post hoc multiple comparisons were used according to the number of groups in the ABA data. Volcano plots displaying DEGs and GO analyses for up‐ and downregulated genes used a negative log FDR of > 1.3 to determine statistical significance (*p < 0*.05).

## 3 Results

### 3.1 Susceptible and Resistant Rats Demonstrate Distinct Behavioral Phenotypes in ABA

**FIGURE 3:**
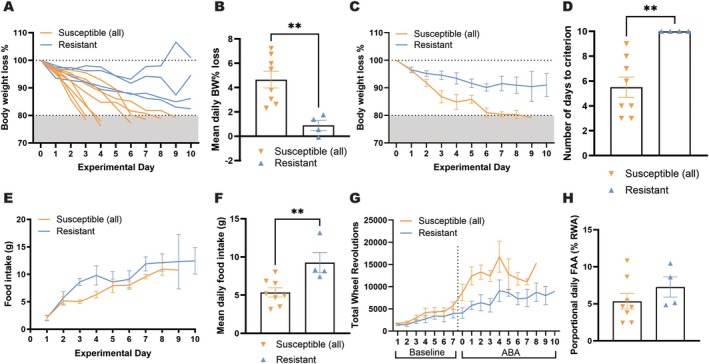
Primary ABA outcome measures for rats Susceptible and Resistant to weight loss. As expected based on susceptibility criteria, Resistant rats were able to maintain body weight above the threshold of 80% baseline body weight for the entire 10‐day period, whereas Susceptible rats were not (A) and demonstrated a more rapid weight loss rate (B–D; B, *t*(10) = 3.640, *p* = 0.0045; C, Experimental day *F*(10,74) = 15.0, *p* < 0.0001; ABA Susceptibility *F*(1,10) = 12.5, *p* = 0.0054; D, *t*(10) = 3.770, *p* = 0.0037). Resistant rats also ate more food within the time window than Susceptible rats during the 10‐day ABA period (E, F; E, Experimental day *F*(9,63) = 9.46, *p* < 0.0001; ABA Susceptibility *F*(1,10) = 2.59, *p* = 0.1384; F, *t*(10) = 3.173, *p* = 0.0099). Overall running wheel activity was significantly higher in Susceptible rats (G; Baseline: Experimental day *F*(6,60) = 9.01, *p* < 0.0001; ABA Susceptibility *F*(1,10) = 1.05, *p* = 0.3288; Interaction *F*(6,60) = 0.420, *p* = 0.8629; ABA: Experimental day *F*(10,63) = 3.39, *p* = 0.0013; ABA Susceptibility *F*(1,10) = 10.9, *p* = 0.0079), contributing to their improved body weight maintenance. However, motivated running in anticipation of food (H, *t*(10) = 1.086, *p* = 0.3029) was not significantly elevated in Resistant rats. Grouped data show mean ± SEM, with individual data point on the bar graphs. **p < 0.01. ABA, Activity‐based anorexia; BW, Body weight; FAA, Food anticipatory activity.

In order to confirm the TRAP expression within mPFC before the onset of ABA exposure, we used immunohistochemistry to validate that expression was restricted to neurons projecting to the AcbSh (Figure 2). In accordance with our previously characterized susceptibility criterion (Foldi et al. 2017; Milton et al. 2021; Milton et al. 2018), Resistant rats showed a slower body weight loss rate and maintained > 80% of their baseline body weight for the entire 10‐day ABA exposure period (Figure 3A–D; B, *p* = 0.0045; C, Experimental day *F*(10,74) = 15.0, *p* < 0.0001; ABA Susceptibility *F*(1,10) = 12.5, *p* = 0.0054; D, *p* = 0.0037). Consistent with their ability to maintain body weight, Resistant rats consumed significantly more during the 90 min of food access throughout the 10‐day period (Figure 3E,F; E, Experimental day *F*(9,63) = 9.46, *p* < 0.0001; ABA Susceptibility *F*(1,10) = 2.59, *p* = 0.1384; F, *p* = 0.0099), but did not demonstrate an increased level of FAA (Figure 3H, *p* = 0.3029), contrary to previous reports (Milton et al. 2021). Resistant rats also maintained a relatively stable and moderate level of daily running wheel activity, whereas Susceptible rats showed a clear activity spike at the onset of food restriction (Figure 3G; ABA: Experimental day *F*(10,63) = 3.39, *p* = 0.0013; ABA Susceptibility *F*(1,10) = 10.9, *p* = 0.007).

### 3.2 Differentially Expressed Genes Align With Data From GWAS and Implicate Both Metabolic and Synaptic Processes in Pathological Weight Loss

Motivated by the complex roles of the cortico‐striatal (mPFC‐AcbSh) pathway during cognitive flexibility versus reinforcement learning in ABA rats, next we investigated whether stable molecular phenotypes (i.e., those that persist after body weight is regained) in this pathway are associated with susceptibility to excessive exercise and weight loss. To examine enduring (and perhaps intrinsic) transcriptomic differences within the mPFC‐AcbSh pathway between the two phenotypes, we harnessed the TRAP technology to selectively isolate ribosome‐bound mRNA from this pathway after body weight recovery (at least to 100% of baseline). This approach was chosen to disambiguate compensatory mechanisms that may occur during acute weight loss from possible circuit‐specific differences underlying genetic susceptibility to ABA.

**FIGURE 4:**
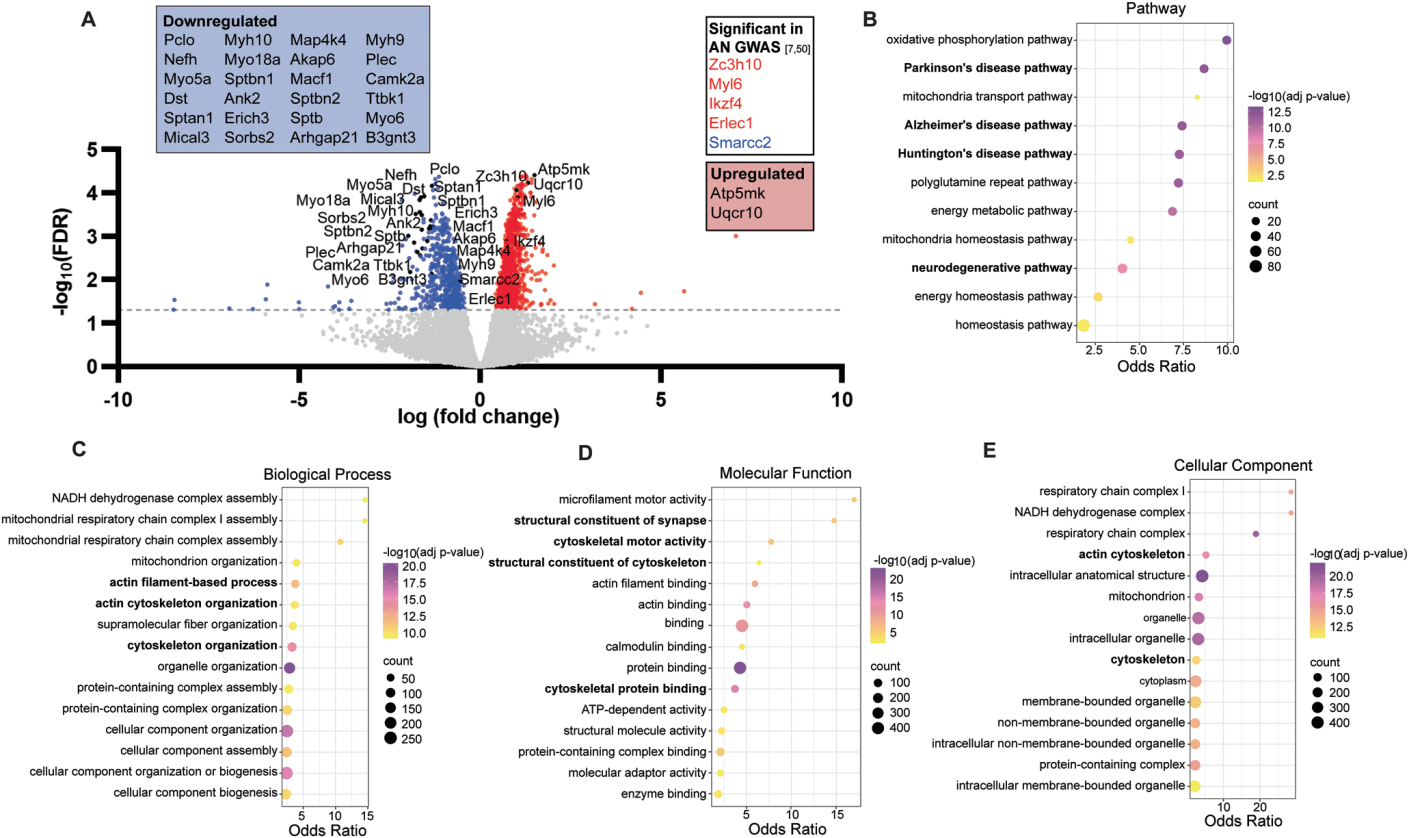
Rats that are Resistant to ABA show distinct gene expression profiles in striatum projecting mPFC neurons compared to Susceptible rats. Volcano plot of 1424 significant differentially expressed genes (DEGs) between Susceptible and Resistant rats (A). Gray dashed line represents a −log_10_(FDR) = 1.3, the threshold for statistical significance (*p < *0.05). The blue dots denote all significantly downregulated genes observed in the Resistant, the red dots denote all significantly upregulated genes in the Resistant group, and gray dots represent genes that differed in expression between groups that did not reach statistical significance. Gene names are indicated for the most significant and stable (i.e., the least between‐sample variability) upregulated (2) or downregulated (24) genes and the 5 genes that are significant in human genome‐wide associated studies for anorexia nervosa (Watson et al. 2019 and Duncan et al. 2017). Pathways that were significantly (corrected *p* value < 0.05) enriched in Susceptible vs. Resistant rats are involved in metabolic functions and neurodegenerative diseases (B). The top 15 biological process (C), molecular function (D), and cellular component (E) gene ontology (GO) terms enriched in DEGs predominantly relate to mitochondrial and cytoskeletal functions. The vertical axis of the bubble plots represents the pathway name or GO term, which is used to describe function of a gene. The horizontal axis represents the Odds Ratio, which is the likelihood of a pathway or GO term to be associated with a set of genes of interest compared to a reference set of genes. A higher odds ratio represents a stronger enrichment of the term or pathway. The color scale for each bubble plot (B–E) indicates different thresholds of the *p* value, whereby a lighter color represents a greater significance. In addition, the bubbles size represents the number of genes observed to be differentially expressed within each pathway or term. Terms with a corrected *p* value < 0.05 and an odds ratio between 1.3 and 40 were included.

edgeR analysis between groups revealed 1424 differentially expressed genes (DEGs) between Susceptible and Resistant rats, highlighting important transcriptional differences associated with Resistance to ABA within this neural circuit (−log_10_FDR > 1.3, Figure 4A). To further understand the biological significance of DEGs, GO analysis revealed that DEGs were significantly enriched in pathways related to oxidative phosphorylation and associated with neurodegenerative diseases, including Parkinson's, Alzheimer's, and Huntington's diseases (Figure 4B). The major molecular and cellular‐related themes that emerged were in genes associated with mitochondrial and cytoskeletal functions, cellular organization, and protein complex assembly (Figure 4C–E). More specifically, among these genes, we found that upregulated genes were mainly involved in metabolic processes (*Atp5mkI*: LogFC = 1.50; *Uqcr10*: LogFC = 1.32) and downregulated genes were involved in cytoskeletal, postsynaptic, and axonal functions (blue box; Figure 4A). Interestingly, five of the DEGs were previously identified in GWAS as AN‐associated genes (Watson et al. 2019). Of these, *Zc3h10*, *Myl6*, *Ikzf4*, and *Erlec1* were upregulated in Resistant compared to Susceptible ABA rats, whereas *Smarcc2* was downregulated in the Resistant group. These genes have functions in thermoregulation (Yi et al. 2019), inflammatory responses (Liu et al. 2023) and endoplasmic reticulum stress (Misiewicz et al. 2013).

### 3.3 A Subgroup of Susceptible Rats Shows an “Intermediate” Behavioral Phenotype

**FIGURE 5:**
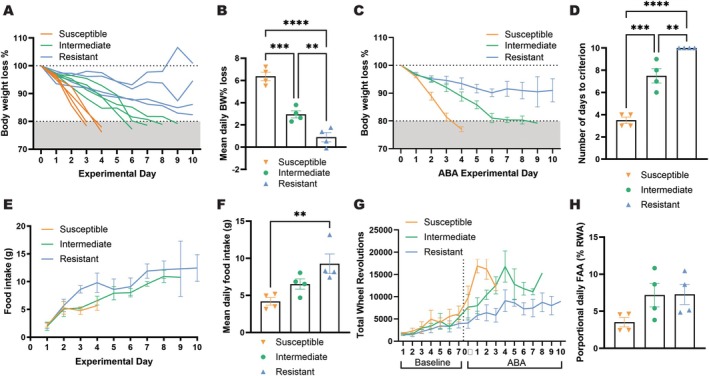
Separation of ABA rats based on trajectory of weight loss reveals an intermediate phenotype. While Susceptible rats lost weight rapidly, Intermediate rats reached the threshold for ABA susceptibility criterion, albeit at a slower rate (A–D, B, *F*(2,9) = 54.3, *p* < 0.0001, Intermediate vs. Susceptible *p* = 0.0003; Intermediate vs. Resistant *p* = 0.0096; Susceptible vs. Resistant *p* < 0.0001; C, Experimental day *F*(10,74) =15.3, *p* < 0.0001, ABA Susceptibility *F*(2,9) = 8.68, *p* = 0.0079; D, *F*(2,9) = 64.5, *p* < 0.0001, Intermediate vs. Susceptible *p* = 0.0002; Intermediate vs. Resistant *p* = 0.0049; Susceptible vs. Resistant *p* < 0.0001) and engaged in feeding at levels between the two other groups (but not significantly different to either; E, F; E, Experimental day *F*(9,63) = 9.06, *p* < 0.0001; ABA Susceptibility *F*(2,9) = 1.22, *p* = 0.3389; F, *F*(2,9) = 7.84, *p* = 0.0107, Resistant > Intermediate *p* = 0.1364, Intermediate > Susceptible *p* = 0.219). Examination of the profiles of daily running wheel activity showed a clear spike in activity at the onset of food restriction (dashed line) for Susceptible rats, whereas Intermediate rats exhibit a more gradual increase in wheel running (G, Baseline: Experimental day *F*(6, 54) = 11.50, *p* < 0.0001; ABA Susceptibility *F*(2, 9) = 0.7325 *p* = 0.5073; Interaction *F*(12,54) = 0.7031, *p* = 0.7415; ABA: Experimental day *F*(10,63) = 3.58, *p* = 0.0008; ABA Susceptibility *F*(2,9) = 8.24, *p* = 0.0092). Interestingly, Resistant and Intermediate rats engaged in the same level of motivated running in anticipation of food (FAA) (H, *F*(2,9) = 2.889, *p* = 0.1074, Intermediate vs. Susceptible *p* = 0.1550, Resistant vs. Intermediate *p* = 0.9984). Grouped data show mean ± SEM, with individual data point on the bar graphs. **p < 0.01, ***p < 0.001, ****p < 0.0001. ABA, activity‐based anorexia; BW, body weight; FAA, food anticipatory activity.

Through our extensive experience with the ABA model, we have noticed phenotypic differences within those rats susceptible to ABA, particularly with respect to the *rate* of body weight loss. We were therefore interested to understand whether differences in weight loss trajectory were related to gene expression within this cortico‐striatal pathway. Here, we distinguish susceptible rats as those that lost weight rapidly (i.e., reached the threshold for ABA susceptibility criterion in < 4 days) and intermediate rats as those that still reached this criterion, albeit at a slower rate (Figure 5A–D; B, Intermediate vs. Susceptible *p* = 0.0003; Intermediate vs. Resistant *p* = 0.0096; Susceptible vs. Resistant *p* < 0.0001; C, *p* = 0.0079, D, Intermediate vs. Susceptible *p* = 0.0002; Intermediate vs. Resistant *p* = 0.0049; Susceptible vs. Resistant *p* < 0.0001). As described above, resistant rats ate significantly more when it was available than susceptible rats (Figure 5E,F; E, Experimental day *F*(9,63) = 9.06, *p* < 0.0001; ABA Susceptibility *F*(2,9) = 1.22, *p* = 0.3389; F, *p* = 0.0084), and intermediate rats consumed food in amounts between the two other groups. Interestingly, resistant and intermediate rats engaged in the same level of food anticipatory activity, and although this was reduced for susceptible rats, the difference was not significant (Figure 5H, Intermediate vs. Susceptible *p* = 0.1550; Resistant vs. Susceptible *p* = 0.1430). Examination of the profiles of daily running wheel activity showed a clear spike in activity at the onset of food restriction (dashed line) for susceptible rats, whereas intermediate rats exhibit a more gradual increase in wheel running and resistant rats maintain a relatively constant rate of exercise (Figure 5G ABA: Experimental day *F*(10,63) = 3.58, *p* = 0.0008; ABA Susceptibility *F*(2,9) = 8.24, *p* = 0.0092). Notably, susceptible and intermediate rats demonstrated no significant differences in body weight recovery in the first 3 days after reaching weight loss criterion, supporting the separation of the two subgroups was specific to the ABA condition (Figure S1).

**FIGURE 6:**
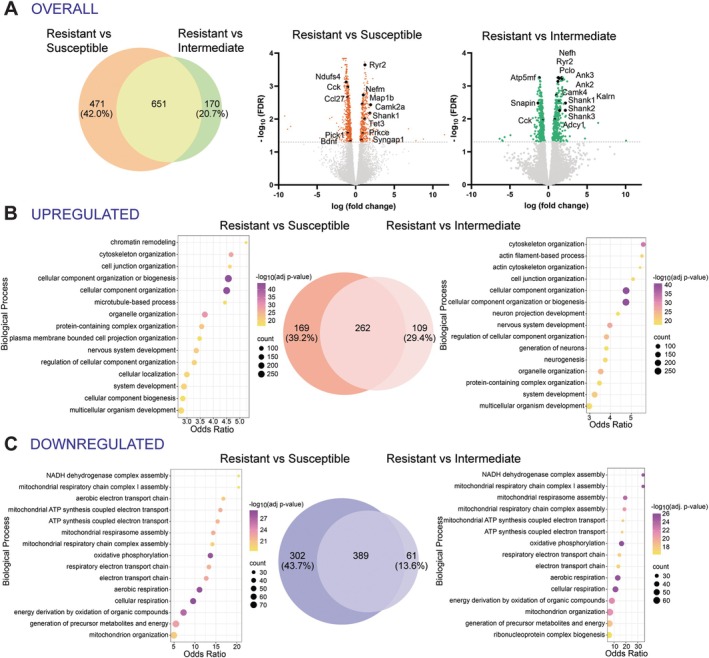
Gene expression profiles for ABA Resistant rats, compared to Susceptible or Intermediate subgroups. Venn diagram shows overlap of differentially expressed genes (DEGs) in Susceptible and/or Intermediate compared to Resistant rats (A), noting a large group of genes (651) are common to both comparisons and that there are fewer DEGs exclusively found in the Resistant vs. Intermediate comparison (170) than the Resistant vs. Susceptible comparison (471). Volcano plots of DEGs for Resistant compared to Susceptible or Intermediate subgroup. The significant DEGs [−log_10_(FDR) > 1.3] are indicated in orange or green, with gene names of interest indicated for genes that are related to neurodevelopmental disorders, synaptic function, satiety, inflammatory processes and feeding behaviors, all processes relevant to the development of ABA in rats and AN in humans. Venn diagram shows overlapping of DEGs that are upregulated in Susceptible (left) or Intermediate (right) rats compared to Resistant rats (B), with 39.2% of upregulated DEGs that are exclusive to Susceptible but not Intermediate. Ten of the Top 15 biological process terms enriched in the upregulated DEGs common between the two comparisons (i.e., Susceptible vs. Resistant and Intermediate vs. Resistant—see bubble plots). With respect to downregulated DEGs, the Venn diagram shows that 43.7% of identified genes are exclusive to the Susceptible vs. Resistant group comparison and do not overlap with those in the Intermediate vs. Resistant group comparison (C). Here, 14 of the Top 15 biological process terms enriched in the downregulated DEGs are common to both groups (see bubble plots), which are involved in metabolic and mitochondrial functions and suggests that these processes are a shared feature of both “weight losing” groups.

With this weight loss separation in mind, we examined the differences between two subgroups of Susceptible rats compared to the Resistant group and identified genes that were differentially expressed between groups as well as those that overlapped. Overall, 651 DEGs were identified as overlapping and substantially fewer DEGs were identified between the Resistant and Intermediate subgroups than those identified between the Resistant and Susceptible, supporting their separation into discrete categories (Figure 6A). Within these DEGs, 170 genes exclusively differed in the Intermediate group, whereas 471 exclusively differed in the Susceptible group, further supporting the subgroup delineation. Moreover, GO analysis revealed zero overlap between the two comparator groups in terms of the top 15 biological processes, with the Resistant and Susceptible rats mostly differing in genes related to metabolic and mitochondrial functions, whereas the Resistant and Intermediate rats were most different by structural constituent and cytoskeletal functions. Specifically, the Intermediate group had upregulated genes associated with neurodevelopmental disorders (e.g., *Shank1‐3*) whereas the Susceptible group was more genetically distinct from the Resistant rats, with upregulation of genes related to synaptic function (e.g., *Camk2a*) and downregulation of genes involved in satiety (e.g., *Cck*), inflammatory processes (e.g., *Ccl27*), and neuronal growth (e.g., *Bdnf*).

We were also interested in examining the up‐and downregulated DEGs between the Susceptible and Intermediate subgroups compared to the Resistant group separately. Similar to the overlap in DEGs, there was broad overlap between the Susceptible and Intermediate comparisons with Resistant rats, in which 262 upregulated genes overlapped (Figure 6B) and 389 downregulated genes overlapped (Figure 6C), with fewer DEGs identified between Resistant and Intermediate subgroups than those identified between Resistant and Susceptible. In addition, 10 of the top 15 biological processes enriched for upregulated genes in both comparisons (Susceptible vs. Resistant and Intermediate vs. Resistant) were the same, and 14 out of the top 15 biological processes enriched for downregulated genes were common to both Susceptible and Intermediate comparisons with Resistant rats.

### 3.4 Susceptible and Intermediate Subgroups Show No Significant Differentially Expressed Genes

To determine whether Susceptible and Intermediate rats showed DEG within this same pathway, we directly compared the profile of these subgroups. Strikingly, no genes were differentially expressed using the statistically corrected value for significance, indicating the two subgroups of “ABA susceptible” rats do not display detectable differences in expression at the single gene level.

## 4 Discussion

In this study, we used a novel viral‐based TRAP technique to reveal transcriptional changes within the cortico‐striatal neuronal circuit that are associated with susceptibility to pathological weight loss in ABA rats. These changes were associated with metabolic, mitochondrial, and neuronal functions and independent of current calorie deficit, since assessment occurred after weight recovery. Upregulated genes in Resistant rats were enriched for mitochondrial function, in line with a recent report of mitochondrial damage leading to oxidative stress in ABA rats (Bhasin et al. 2023), while downregulated genes were involved in cytoskeletal, postsynaptic, and axonal functions. Overall, these findings point to *decreased excitability of the mPFC‐AcbSh circuits* as a molecular signature that may confer resistance to weight loss. This is consistent with our previous study showing that chemogenetic *suppression* of this pathway caused resistance to pathological weight loss during ABA (Milton et al. 2021) and the evidence of elevated activity of PFC regions in humans weight recovered from AN (Ehrlich et al. 2015).

GWAS provide an excellent starting point for understanding the genetic architecture of complex psychiatric disorders. Our study builds upon GWAS findings by investigating how gene expression (i.e., the “message”) is altered, rather than only the DNA (i.e., the “code”). When comparing the outcomes of the present study to genetic changes reported in the human literature, it is important to note that our study did not compare ABA rats to controls, but rather asked the question: *Among animals exposed to the exact same ABA protocol, what intrinsic biological differences exist between those that develop severe symptoms* versus *those that remain relatively resilient?* Thus, the directionality of effects that are typically defined relative to a physiological baseline in humans needs to be interpreted with this in mind. Nevertheless, one risk gene identified in the largest GWAS investigation in AN cases (Watson et al. 2019), *Erlec1*, was significantly downregulated in Susceptible compared to Resistant rats, but this was not significant when comparing Intermediate to Resistant rats, suggesting that differences observed in weight loss trajectories might involve dysregulated protein metabolism. Unfolded or misfolded proteins in the endoplasmic reticulum (ER) are recognized by lectins including *Erlec1*, and an impairment in this recognition process could disrupt ER homeostasis and cause tissue inflammation and endocrine dysregulation (Lemmer et al. 2021). Notably, *Erlec1* demonstrates reliable patterns of under‐expression in central nodes within fear and reward circuits in AN, suggesting that genes linked with AN and vulnerability to rapid weight loss in ABA may confer risk by altering processes related to fear‐ and reward‐learning (Murray et al. 2023), consistent with reports of persistent alterations in these processes after weight gain in AN (Murray et al. 2018) and with the role of this cortico‐striatal circuit in punishment learning (conditioned suppression) (Piantadosi et al. 2020).

In addition, genes associated with feeding behavior and reward processing, including *Bdnf*, were significantly downregulated in Susceptible rats compared to Resistant rats, but not for the Intermediate weight losing subgroup. *Bdnf* mRNA expression is reduced in mPFC by scheduled feeding in mice (Ho et al. 2016) and BDNF serum concentration is reduced in patients with AN at acute and early recovery stages (Borsdorf et al. 2021; Brandys et al. 2011). These data support the possibility that differences in weight loss trajectory in ABA could result from altered reward‐related feeding behavior (Foldi et al. 2017). Interestingly, despite the evidence that BDNF itself has anorectic effects, serum levels of BDNF increase continuously over long‐term recovery in AN, which may represent an enduring effect of prior weight loss or a reinstatement of pre‐existing elevated levels (Borsdorf et al. 2021). Intriguingly, the *Cck* gene, encoding the peptide hormone cholecystokinin (CCK), was downregulated in both Susceptible and Intermediate rats, compared to Resistant rats. The direction of effect here seems counterintuitive when considering the role of CCK in signaling satiety (Moran 2000); however, it aligns with the observation of reduced circulating CCK in patients with AN (Baranowska et al. 2000). Thus, considering gene expression reported here is restricted to the cortico‐stratal circuitry, the downregulation we show may relate to the way that “higher order” cortical neurons that express CCK mRNA in the rat (Burgunder and Young 3rd 1990) regulate appetite, through behavioral mechanisms such as attention and impulse control.

The view that separate genetic profiles are involved in the genesis of rapid versus slower weight loss in ABA rats is supported to some extent by the GSEA. Focusing on the top significantly enriched pathways associated with the Susceptible or Intermediate ABA phenotype (see Figure S2), it is clear that dysregulation of genes associated with mitochondrial function (transport, dynamics and homeostasis) is common to both “weight losing” groups, whereas dysregulation of genes involved in neurodegenerative disease pathways was specific to rapid weight losers (i.e., Susceptible) and not significantly associated with the Intermediate phenotype. Individuals that respond to ABA conditions with rapid weight loss could be separated in future studies to investigate whether common neurochemical disturbances seen in Parkinson's Disease (i.e., loss of dopamine neurons) or Alzheimer's Disease (i.e., loss of acetylcholine neurons) play a role in the development or maintenance of AN‐like behaviors. A better understanding of these synergies could lead to more effective treatments, in light of the fact that pharmacotherapies approved for neurodegenerative diseases, such as donepezil (Aricept), have been proposed as a feasible possibility for treating AN (Bucay 2009), and recent advances in understanding the AN‐relevant mechanisms in mouse models (Favier et al. 2020). Interestingly, although the Enriched Pathways and Biological Processes were distinct when comparing Susceptible or Intermediate rats to Resistant rats, the Cellular Components and MFs identified as significantly dysregulated were largely overlapping (see Figures S3 and S4). Consistent with the significant co‐morbidity between AN and psychiatric disorders including depression, anxiety, substance use, and obsessive‐compulsive disorder (OCD) (Salbach‐Andrae et al. 2008), genetic correlations between these have also been revealed via single‐nucleotide polymorphism (SNP)‐based methods. In particular, AN has the strongest (positive) genetic correlation with OCD (Watson et al. 2019) and shares similar alterations in PFC gene expression (Song et al. 2021), suggesting a common dysregulated functional pathway. Direct evidence of genome‐wide significant risk that is shared between AN and OCD has not been identified; however, several genes have been suggested to drive the association, including *Lrrc16a* and *Kit* (Yilmaz et al. 2020), both involved in cell signaling pathways within the gastrointestinal tract. Moreover, 109 pleiotropic loci were identified in a cross‐disorder GWAS of psychiatric disorders (including AN and OCD), in which the *Dcc* gene, the product of which plays a key role in guiding axonal growth during development, was significantly associated with all eight psychiatric disorders (Lee et al. 2019). In addition, this study identified common genetic associations between AN and major psychiatric disorders including schizophrenia, bipolar disorder, and major depression, as well as psychological traits including mood instability, neuroticism, and intelligence, of which several (*Csnk2b, Ctsa, Gpx1, P4htm and Tcf7l2*) were differentially expressed by the Susceptible and Resistant rats, suggesting that the ABA model might recapitulate some of the genetic variants associated with greater predisposition to AN and other psychiatric traits. Despite the fact that the most translatable feature of the ABA model is the development of compulsive exercise, which is an intermediate phenotype of shared genetic risk for both OCD and AN (Yilmaz et al. 2023), the putative genes that link AN to OCD were not found to be differentially dysregulated in the present study. This could be due to the fact that specific risk loci shared by AN and OCD have yet to be pinpointed (Bulik et al. 2022) or that excessive exercise is driven not by altered mPFC‐AcbSh pathway activity but by disrupted function in other cortico‐striatal pathways, for example, those terminating in the dorsal striatum (Conn et al. 2024).

## 5 Limitations and Future Directions

While our study provides novel insight into transcriptomic signatures associated with vulnerability to ABA, several limitations constrain the interpretation and immediate translational impact of our findings. Chief among these is that the current work stops short of establishing causality between differentially expressed genes (DEGs) and AN‐like phenotypes. Although our results identify promising molecular targets within PFC–AcbSh projecting neurons, future studies should employ gene manipulation strategies (e.g., viral overexpression or knockdown) to determine whether altering expression of candidate DEGs can modulate ABA susceptibility. Specifically, manipulating expression in vulnerable rats to promote resilience (or vice versa) would allow for a more definitive demonstration of causal relevance. Additionally, pharmacological validation of identified pathways, if feasible, could provide important preclinical support for novel therapeutic strategies. Despite this, our results support the use of the ABA model in investigating causal genetic factors to AN and provide directions for the development of translationally relevant genetic models of AN.

A related limitation is the absence of molecular validation for key DEGs. As with all unbiased RNA sequencing techniques, our approach carries an inherent risk of false positives and negatives. Although we applied appropriate statistical correction and prioritized biologically plausible targets for discussion, confirmatory experiments are warranted. In particular, in situ hybridization or immunohistochemistry in conjunction with the retrograde labeling approach used here would help establish spatial and cell‐type specificity of gene expression changes within mPFC–NAc projection neurons. With respect to behavioral controls, we clarify that our study design explicitly sought to contrast rats that were susceptible versus resistant to ABA. That is, within the same experimental conditions of food restriction and running wheel access, the tendency to lose weight differentiates vulnerable rats from those who maintain weight. In this context, susceptible animals serve as the relevant behavioral control and demonstrate the same trajectory of weight loss and excessive exercise as we have reported numerous times (Huang et al. 2023; Milton et al. 2021; Milton et al. 2018; Milton et al. 2022). Nonetheless, the absence of control comparisons incorporating naïve, food‐restricted only (no running) and/or ad libitum fed wheel running groups prevents our results from addressing whether gene expression profiles in rats resistant to ABA are more similar to control than those observed in susceptible rats. Inclusion of these groups could define “true” up‐ or down‐regulation relative to normal conditions, instead of relative shifts between ABA‐associated phenotypes. In addition, although all animals in this study had regained their initial body weight at the collection endpoint, there was some variability in average body weight percentages (107.5% vs. 101.7% of baseline for susceptible versus resistant groups), which may potentially contribute to differences in gene expression at this point in time.

In conclusion, this study suggests that weight loss in ABA results from hyperexcitability in this cortico‐striatal pathway, which is “normalized” in rats resistant to weight loss. The increased expression of axonal and synaptic genes could indicate that too much neuronal activity in this circuit may cause maladaptive behavior. Future studies should aim to validate this interpretation with circuit‐based studies that include markers of pre‐ and post‐synaptic densities, in which the hypothesis might be that a greater “synaptic load” would be colocalized with the mPFC‐AcbSh projecting cells in ABA Susceptible compared to Resistant rats. In addition, the intermediate phenotype involves unique transcriptional signatures that are neither “halfway” between resistant and susceptible states nor overlapping with either extreme group. This indicates that ABA vulnerability may involve multiple, independent biological pathways rather than a single progressive mechanism. The intermediate group may utilize different compensatory mechanisms or stress responses that partially protect against severe weight loss. This has important implications for understanding eating disorder heterogeneity, suggesting that different biological subtypes of vulnerability may exist, each potentially requiring distinct therapeutic approaches.

## Author Contributions

**K. Huang:** investigation, writing – original draft, visualization, writing – review and editing, formal analysis, data curation. **M. A. Magateshvaren Saras:** investigation, methodology, formal analysis. **K. Conn:** investigation, writing – review and editing, supervision. **E. Greaves:** investigation, methodology. **F. Reed:** writing – review and editing. **S. Tyagi:** investigation, methodology, formal analysis, data curation. **H. Munguba:** methodology, validation, visualization, writing – review and editing. **C. J. Foldi:** conceptualization, funding acquisition, writing – original draft, writing – review and editing, project administration, supervision, resources, data curation.

## Conflicts of Interest

The authors declare no conflicts of interest.

## Supporting information


**Table S1:** Quality report for selected samples. Note that while some samples (in bold) have lower than normal RIN values, the way in which the reads were collected indicate that this was caused by fragmentation (from bead‐based physical immunoprecipitation) rather than by degradation of the samples (Trevor Wilson, *personal communication*).
**Figure S1:** Recovery profile for Susceptible, Intermediate and Resistant subgroups in the first 3 days after reaching weight loss criterion. (A, B) Susceptible and Intermediate rats regained body weight at a similar rate (B, *t*(6) = 0.19890, *p* = 0.849). (E, F) The Intermediate rats consumed more food during the first day of recovery compared to the Susceptible rats, demonstrating greater mean daily food intake (F, *t*(6) = 3.269, *p* = 0.017). (I, J) Susceptible and Intermediate subgroups showed no significant difference in running wheel activity during recovery (J, *t*(6) = 0.442, *p* = 0.674). (C, D; G, H; K, L) To demonstrate recovery profile for Resistant rats, susceptible and resistant rats were selected from our previous work to match the body weight percentage observed in the animals sequenced in this current study (Huang et al. 2023). (C, D) While resistant rats reached a higher absolute level of body weight than the susceptible rats during recovery, they showed no significant difference in body weight regain rate (D, *t*(6) = 2.059, *p* = 0.0851). (G, H) Consistent with the greater level of body weight, the resistant rats demonstrated greater daily food intake and mean daily food intake than the susceptible rats (H, *t*(6) = 2.668, *p* = 0.0371). (K, L) The resistant and susceptible rats had a similar level of running wheel activity during recovery (L, *t*(5) = 1.309, *p* = 0.2474). Grouped data show mean ± SEM, with individual data point on the bar graphs. *p < 0.05.
**Figure S2:** Gene ontology terms and pathways enriched in differentially expressed genes for Resistant compared to Susceptible or Intermediate subgroups. The top 15 significantly (corrected *p* value < 0.05) enriched biological process (A), cellular components (B), molecular functions (C) gene ontology (GO) terms and pathways (D) are shown in bubble plots, with Venn diagrams depicting the specific overlap of these GO terms or pathways that were associated with the Susceptible (orange) or Intermediate (green) subgroups compared to Resistant rats. (A) The top 15 significant biological process enriched were entirely distinct for the comparisons between Susceptible versus Resistant and Intermediate versus Resistant groups. Differentially expressed genes (DEGs) in the Susceptible subgroup were most significantly associated with mitochondrial function and energy metabolism, whereas DEGs in Intermediate subgroup were mainly associated with cytoskeletal structure and protein interactions. (B) Cellular component terms included intracellular anatomical structure, organelle, intracellular organelle and cytoplasm that were common to both subgroups (see bold text term names), whereas mitochondrial structure and function are significantly dysregulated in Susceptible subgroup and cytoskeletal and neuronal structures are associated with the Intermediate subgroup. (C) The top 15 molecular function terms largely overlap between the two subgroups (11/15; cytoskeletal dynamics, synaptic function and protein binding), however, pre‐ and postsynaptic function differed between the comparison groups, whereby presynaptic function was exclusively associated with the Susceptible subgroup and postsynaptic function exclusively within the Intermediate subgroup (see bold terms). (D) Pathways associated mitochondrial function (transport, dynamics and homeostasis) are common to both Susceptible and Intermediate subgroups, whereas pathways involved in neurodegenerative diseases (Parkinson's, Huntington's and Alzheimer's Disease) were only seen when comparing the Susceptible subgroup to Resistant rats.
**Figure S3:** Gene ontology terms enriched in upregulated differentially expressed genes for Resistant compared to Susceptible or Intermediate subgroups. The top 15 significantly (corrected *p* value < 0.05) enriched biological process (A), cellular component (B), molecular function (C) gene ontology (GO) terms are shown in bubble plots, with Venn diagrams depicting the specific overlap of these GO terms that were associated with the Susceptible (pink) or Intermediate (light pink) subgroups compared to Resistant rats. (A) 10 of the top 15 biological processes were enriched for upregulated genes in both Susceptible or Intermediate subgroups, including cellular organization and development, whereas actin cytoskeleton dynamics and neuronal function terms were specific to upregulated genes in the Intermediate subgroup, and the chromatin remodeling and microtubule‐based process terms were specific to the Susceptible rats (bold terms). (B) Cellular components terms largely overlapped (14/15) involving synaptic functions, cellular organization and intracellular structures, however the somatodendritic compartment term was specific to the Susceptible subgroup comparison and the neuron to neuron synapse term was specific to the Intermediate subgroup comparison (bold terms). (C) Upregulated genes in both subgroups are commonly associated with binding functions (bold), although the specificity of binding terms was a mix of overlapping and distinct.
**Figure S4:** Gene ontology terms enriched in downregulated differentially expressed genes for Resistant compared to Susceptible or Intermediate subgroups. Top 15 significantly (corrected *p* value < 0.05) enriched biological process (A), cellular component (B), molecular function (C) gene ontology (GO) terms are shown in bubble plots, with Venn diagrams depicting the specific overlap of these GO terms that were associated with the Susceptible (blue) or Intermediate (violet) subgroups compared to Resistant. (A) Biological processes mostly overlap between two subgroup comparisons (14/15), as do (B) the cellular components terms (12/15) and (C) molecular processes (12/15). The interesting differences between downregulated genes within these comparisons include (A) aerobic electron transport chain, (B) organelle envelope and (intracellular) membrane‐bound organelle, (C) 2 iron 2 sulfur cluster binding, electron transfer activity and enzyme binding downregulated in Susceptible; and (A) ribonucleoprotein complex biogenesis, (B) respiratory chain complex, NADH dehydrogenase complex and oxidoreductase complex, (C) proton channel activity, ubiquitin‐protein transferase inhibitor and regulator activity downregulated in Intermediate (see bold text).

## Data Availability

The data that support the findings of this study are available from the corresponding author upon reasonable request.
