# Supplementary material for: Toward a Genetic Signature of Resistance to Activity‐Based Anorexia in Striatal Projecting Cortical Neurons
Source: Int J Eat Disord. 2025 Sep 5;58(12):2292–305. doi: 10.1002/eat.24538 (PMC12703224; doi:10.1002/eat.24538)
Supplement: Supplementary file 1 — Table S1: Quality report for selected samples. Note that while some samples (in bold) have lower than normal RIN values, the way in which the reads were collected indicate that this was caused by fragmentation (from bead‐based physical immunoprecipitation) rather than by degradation of the samples (Trevor Wilson, personal communication). Figure S1: Recovery profile for Susceptible, Intermediate and Resistant subgroups in the first 3 days after reaching weight loss criterion. (A, B) Susceptible and Intermediate rats regained body weight at a similar rate (B, t(6) = 0.19890, p = 0.849). (E, F) The Intermediate rats consumed more food during the first day of recovery compared to the Susceptible rats, demonstrating greater mean daily food intake (F, t(6) = 3.269, p = 0.017). (I, J) Susceptible and Intermediate subgroups showed no significant difference in running wheel activity during recovery (J, t(6) = 0.442, p = 0.674). (C, D; G, H; K, L) To demonstrate recovery profile for Resistant rats, susceptible and resistant rats were selected from our previous work to match the body weight percentage observed in the animals sequenced in this current study (Huang et al. 2023). (C, D) While resistant rats reached a higher absolute level of body weight than the susceptible rats during recovery, they showed no significant difference in body weight regain rate (D, t(6) = 2.059, p = 0.0851). (G, H) Consistent with the greater level of body weight, the resistant rats demonstrated greater daily food intake and mean daily food intake than the susceptible rats (H, t(6) = 2.668, p = 0.0371). (K, L) The resistant and susceptible rats had a similar level of running wheel activity during recovery (L, t(5) = 1.309, p = 0.2474). Grouped data show mean ± SEM, with individual data point on the bar graphs. *p < 0.05. Figure S2: Gene ontology terms and pathways enriched in differentially expressed genes for Resistant compared to Susceptible or Intermediate subgroups. The top 15 significantly (co [file EAT-58-2292-s001.docx]

**Supplementary Table 1. Quality report for selected samples.** Note that while some samples (**in bold**) have lower than normal RIN values, the way in which the reads were collected indicate that this was caused by fragmentation (from bead-based physical immunoprecipitation) rather than by degradation of the samples (Trevor Wilson, *personal communication*).

| **Sample number** | **Days to <80% BW criterion** | **Group** | **RNA Integrity Number (RIN)** | **BA conc [ng/µl]** | **i7 Index** | **Reads (M)** |
| --- | --- | --- | --- | --- | --- | --- |
| 1 | / | Resistant | 6.5 | 1.68 | TAAGGCGA | 34.1 |
| 3 | / | Resistant | 7.2 | 2.18 | TAAGGCGA | 88.5 |
| 4 | 4 | Susceptible | 7.4 | 1.59 | AGGCAGAA | 29.0 |
| 8 | 4 | Susceptible | 6.2 | 3.04 | TCCTGAGC | 20.8 |
| 9 | 6 | Intermediate | 7.7 | 10.75 | GGACTCCT | 27.0 |
| 11 | / | Resistant | 6.5 | 3.02 | TAGGCATG | 21.6 |
| 12 | 7 | Intermediate | 9.6 | 3.74 | CTCTCTAC | 38.8 |
| 15 | 3 | Susceptible | **3.4** | 1.81 | CGAGGCTG | 29.6 |
| 16 | 3 | Susceptible | **3.0** | 1.60 | AAGAGGCA | 109.9 |
| 23 | 8 | Intermediate | **3.6** | 5.97 | GTAGAGGA | 25.2 |
| 26 | 9 | Intermediate | **3.8** | 3.46 | GCTCATGA | 56.0 |
| 27 | / | Resistant | **4.8** | 0.51 | ATCTCAGG | 42.3 |

**Supplementary Methods:**

Immunohistochemical validation of TRAP viral expression

To validate the expression of the EGFPL10a (TRAP) construct, stereotaxic injections were performed as described. Two weeks later, rats (n=2) were euthanized with sodium pentobarbitone (Lethabarb 150mg/kg; Virbac, AU) and transcardially perfused with 200mL 0.9% saline followed by 200mL 4% paraformaldehyde in phosphate buffer. Brains were excised and postfixed in 4% paraformaldehyde in phosphate buffer overnight at 4°C, followed by submersion in 30% sucrose in phosphate buffer for 4 days. Brains were then sectioned at 35µm using a cryostat (CM1860; Leica Biosystems) and collected in a 1:4 series. A series of sections were immunolabeled with chicken anti-GFP (1:1000, Abcam) and guinea pig anti-NeuN (1:1000, Synaptic Systems) primary antibodies overnight at room temperature, followed by 90-minute incubation in Alexa-fluor conjugated secondary antibodies (1:1000, Alexa-fluor 488, Jackson ImmunoResearch; 1:1000, Alexa-fluor 594, Abcam) and 5-minute incubation in DAPI (1:5000, Sigma-Aldrich). Sections were imaged on a widefield microscope (Thunder Imager Live Cell & 3D Assay, Leica Microsystems, Germany).

Translating Ribosome Affinity Purification (TRAP) assay

To prepare the tissue for extraction of EGFP-tagged polysomes, the tissue was homogenised with a hand pestle mixer in lysis buffer [10 mM HEPES-KOH, 150 mM KCl, 5 mM MgCl2, 0.5 mM DTT, 100 µg/ml CHX, RNase inhibitor (RNasin, Promega, 40u/µl) and protease inhibitors (Roche, 1 tablet/ml)], followed by centrifugation at 2000g at 4°C for 10 minutes. The supernatant was then incubated with NP-40 working solution and DHPC on ice for 5 minutes, followed by centrifugation at 13,000 g for 10 minutes at 4°C. Following centrifugation, polysomes were immunoprecipitated from the supernatant by overnight incubation at 4°C with anti-GFP antibody-labelled Dynabeads (Invitrogen, California, USA). The bound RNA was collected with a magnetic rack and washed with a KCl wash buffer (10 mM HEPES-KOH, 350 mM KCl, 5 mM MgCl2, 1% NP-40, 0.5 mM DTT, 100 µg/ml CHX), followed by RNA extraction using the RNeasy Micro Kit (Qiagen, The Netherlands).

## RNA Sequencing

Total RNA samples underwent quality control by bioanalyser and the resulting library pool was QC’d by Qubit, Bioanalyzer and qPCR. It had a mean size of 361bp at a concentration of 9.9nM. Sequencing was performed using NextSeq2000 on a P2 100cycle kit (cDNA reads generated 101nt), with read coverage up to 400M reads per run. A novel RNA-Seq pipeline (Version 01/09/2021) was used on the final library pool, loaded at 1000pM (based upon size adjusted qPCR) for on-board denaturation and clustering (Illumina Protocol; 1000000109376 v3 Nov2020) which resulted in a loading metric of 98.42 and 88.95% >Q30. 541.2 reads passed filter and 464 Million reads were assigned to indexes. Briefly, index is added during initial pA priming and pooled samples amplified using template switching oligo. P5 is added by tagmentation by Nextera transposase and PCR. On-board denaturing and clustering were conducted using 1000pM of library pool. Parsing and base calling was conducted (Dragen BCLConvert 3.7.4) and showed that Sample 22-2449 (Resistant) had the lowest amount of sample with 6.5ng total RNA. Since all samples are pooled for the multiplex method, this amount was used as starting amount for all samples.

**Supplementary Information: Definition of Gene Ontology terms**

Gene ontology (GO) describes gene functions in three aspects: Molecular Function, Cellular Component, and Biological Process. Molecular function describes the activity of the gene product at molecular level. Cellular component describes the location where the activity occurs in the cell. Biological process represents biological events contributed by molecular processes.

**
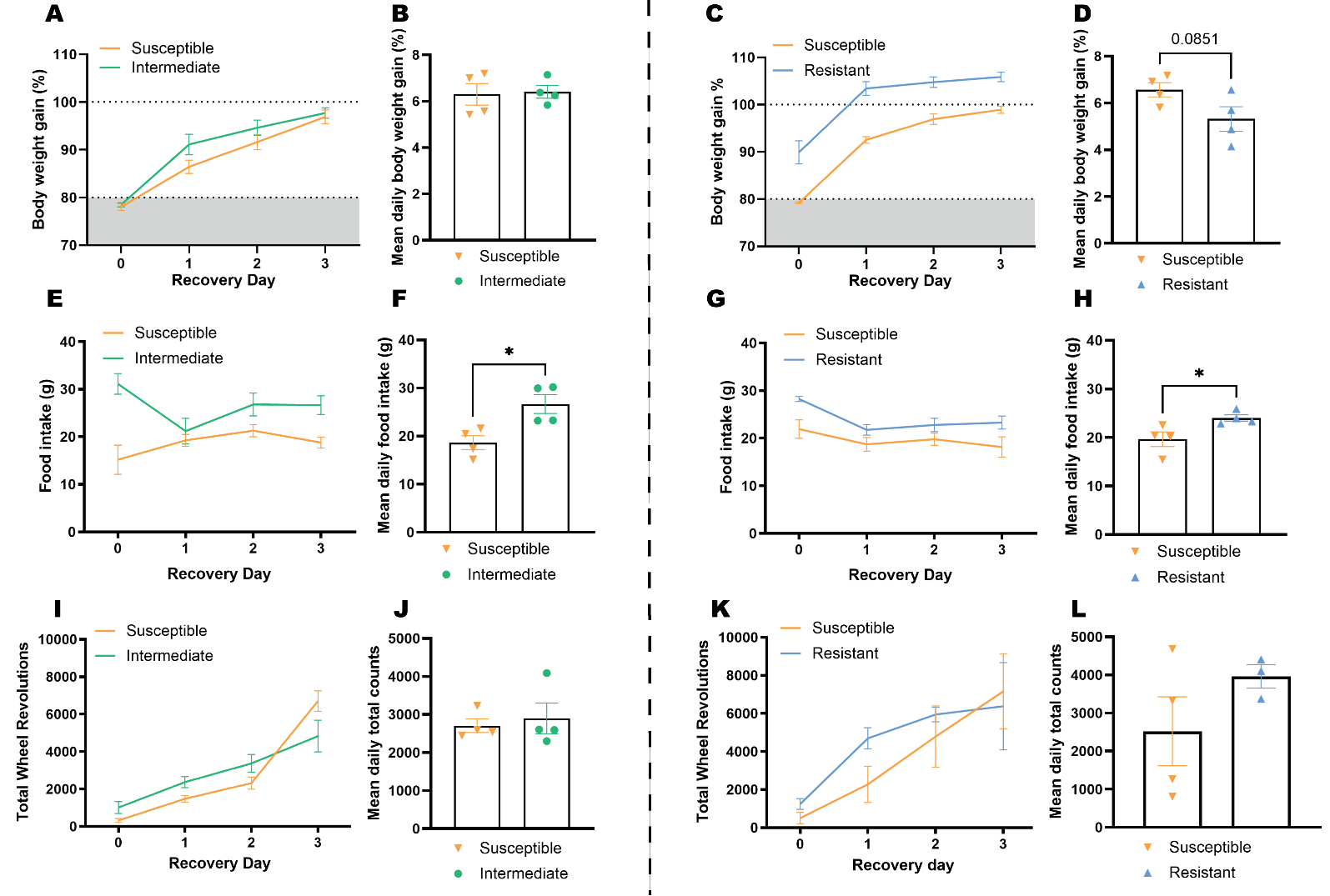
**

**Supplementary Figure 1**. **Recovery profile for Susceptible, Intermediate and Resistant subgroups in the first 3 days after reaching weight loss criterion. (A-B)** Susceptible and Intermediate rats regained body weight at a similar rate [**B,** *t*(6) = 0.19890, *p* =0.849]. **(E-F)** The Intermediate rats consumed more food during the first day of recovery compared to the Susceptible rats, demonstrating greater mean daily food intake [**F,** *t*(6) = 3.269, *p* = 0.017]. **(I-J)** Susceptible and Intermediate subgroups showed no significant difference in running wheel activity during recovery [**J,** *t*(6) = 0.442, *p* = 0.674]. **(C-D; G-H; K-L)** To demonstrate recovery profile for Resistant rats, susceptible and resistant rats were selected from our previous work to match the body weight percentage observed in the animals sequenced in this current study (Huang et al., 2023). **(C-D)** While resistant rats reached a higher absolute level of body weight than the susceptible rats during recovery, they showed no significant difference in body weight regain rate [**D,** *t*(6) = 2.059, *p* = 0.0851]. **(G-H)** Consistent with the greater level of body weight, the resistant rats demonstrated greater daily food intake and mean daily food intake than the susceptible rats [**H,** *t*(6) = 2.668, *p* = 0.0371]. **(K-L)** The resistant and susceptible rats had a similar level of running wheel activity during recovery [**L,** *t*(5) = 0.1.309, *p* = 0.2474]. Grouped data show mean ± SEM, with individual data point on the bar graphs. * p < 0.05.

**
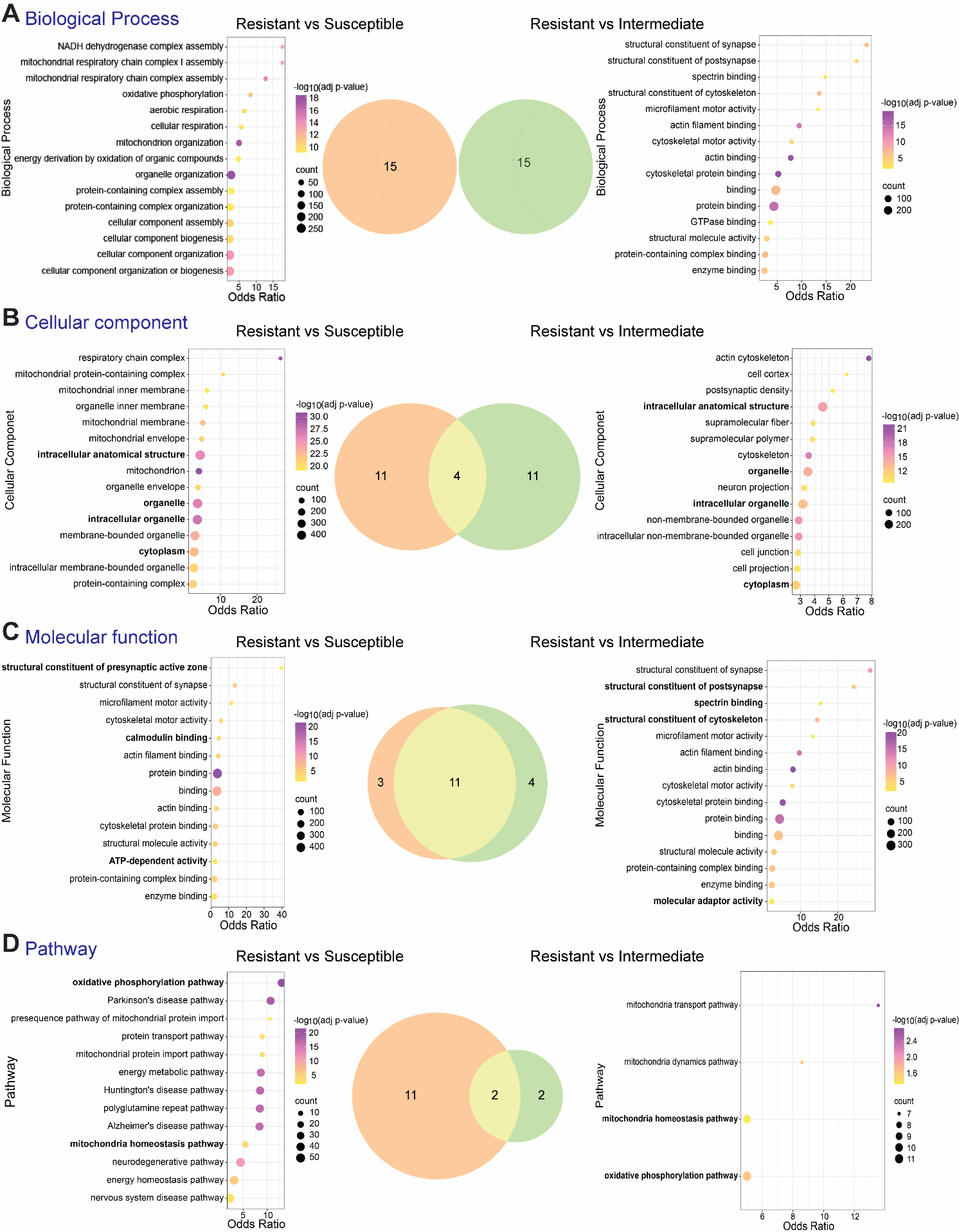
**

**Supplementary Figure 2**. **Gene ontology terms and pathways enriched in differentially expressed genes for Resistant compared to Susceptible or Intermediate subgroups.** The top 15 significantly (corrected *p*-value < 0.05) enriched biological process (**A**), cellular components (**B**), molecular functions (**C**) gene ontology (GO) terms and pathways (**D**) are shown in bubble plots, with Venn diagrams depicting the specific overlap of these GO terms or pathways that were associated with the Susceptible (orange) or Intermediate (green) subgroups compared to Resistant rats. (**A**) The top 15 significant biological process enriched were entirely distinct for the comparisons between Susceptible versus Resistant and Intermediate versus Resistant groups. Differentially expressed genes (DEGs) in the Susceptible subgroup were most significantly associated with mitochondrial function and energy metabolism, whereas DEGs in Intermediate subgroup were mainly associated with cytoskeletal structure and protein interactions. (**B**) Cellular component terms included intracellular anatomical structure, organelle, intracellular organelle and cytoplasm that were common to both subgroups (see bold text term names), whereas mitochondrial structure and function are significantly dysregulated in Susceptible subgroup and cytoskeletal and neuronal structures are associated with the Intermediate subgroup. (**C**) The top 15 molecular function terms largely overlap between the two subgroups (11/15; cytoskeletal dynamics, synaptic function and protein binding), however, pre- and postsynaptic function differed between the comparison groups, whereby presynaptic function was exclusively associated with the Susceptible subgroup and postsynaptic function exclusively within the Intermediate subgroup (see bold terms). (**D**) Pathways associated mitochondrial function (transport, dynamics and homeostasis) are common to both Susceptible and Intermediate subgroups, whereas pathways involved in neurodegenerative diseases (Parkinson’s, Huntington’s and Alzheimer’s Disease) were only seen when comparing the Susceptible subgroup to Resistant rats.


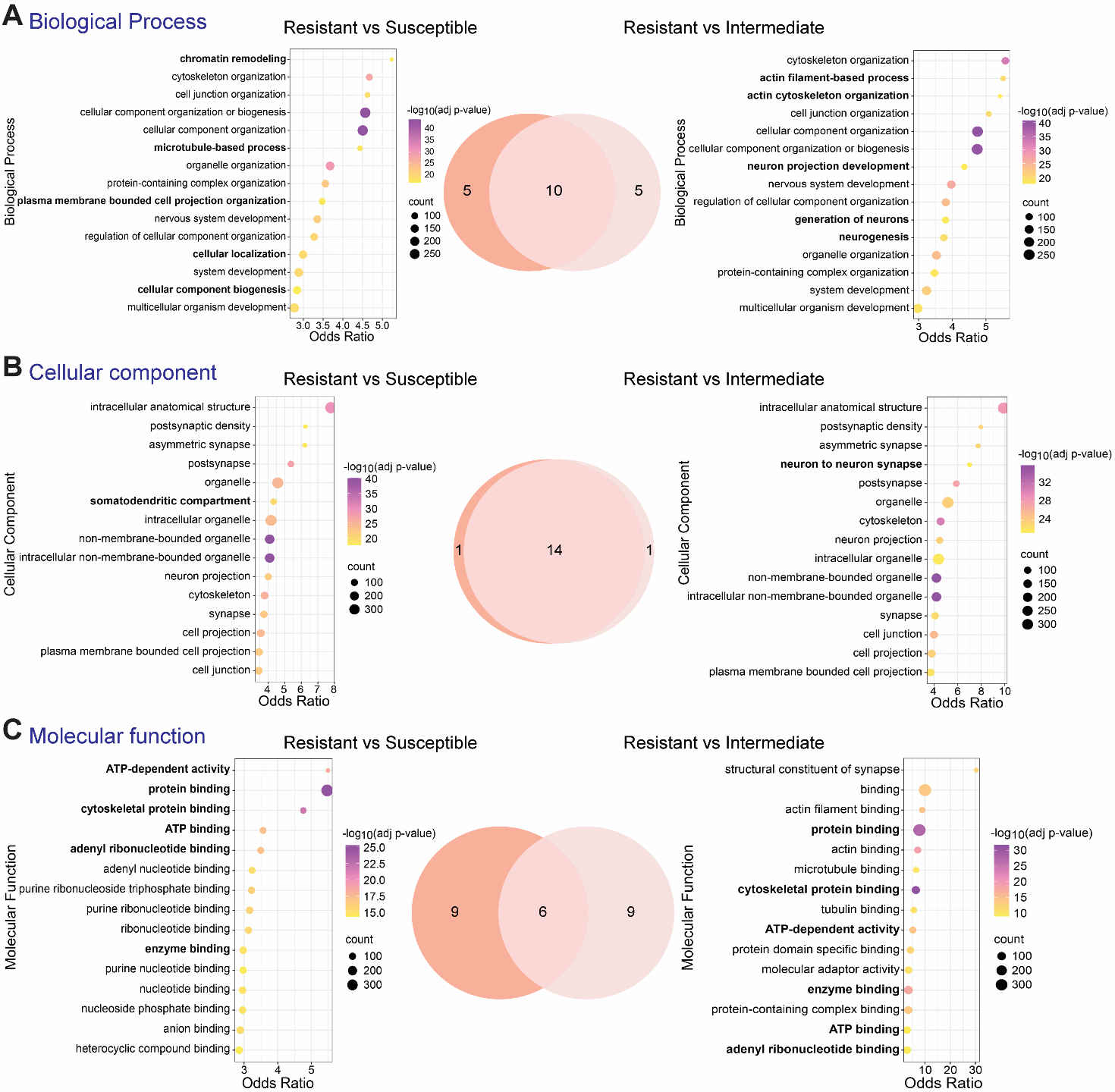


**Supplementary Figure 3**. **Gene ontology terms enriched in upregulated differentially expressed genes for Resistant compared to Susceptible or Intermediate subgroups.** The top 15 significantly (corrected *p*-value < 0.05) enriched biological process (**A**), cellular component (**B**), molecular function (**C**) gene ontology (GO) terms are shown in bubble plots, with Venn diagrams depicting the specific overlap of these GO terms that were associated with the Susceptible (pink) or Intermediate (light pink) subgroups compared to Resistant rats. (**A**) 10 of the top 15 biological processes were enriched for upregulated genes in both Susceptible or Intermediate subgroups, including cellular organization and development, whereas actin cytoskeleton dynamics and neuronal function terms were specific to upregulated genes in the Intermediate subgroup, and the chromatin remodelling and microtubule-based process terms were specific to the Susceptible rats (bold terms). (**B**) Cellular components terms largely overlapped (14/15) involving synaptic functions, cellular organisation and intracellular structures, however the somatodendritic compartment term was specific to the Susceptible subgroup comparison and the neuron to neuron synapse term was specific to the Intermediate subgroup comparison (bold terms). (**C**) Upregulated genes in both subgroups are commonly associated with binding functions (bold), although the specificity of binding terms was a mix of overlapping and distinct.

**
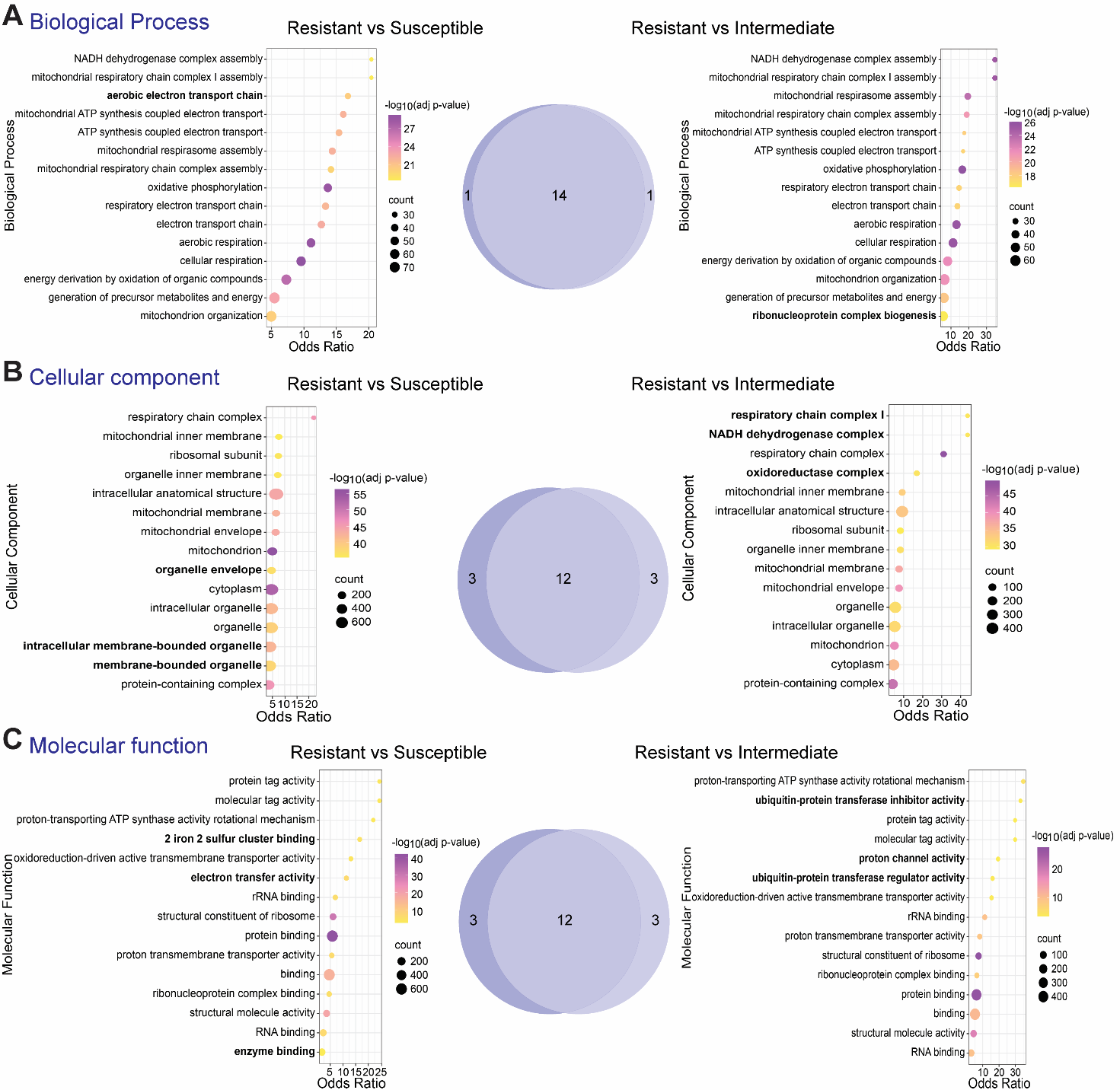
Supplementary Figure 4**. **Gene ontology terms enriched in downregulated differentially expressed genes for Resistant compared to Susceptible or Intermediate subgroups.** Top 15 significantly (corrected *p*-value < 0.05) enriched biological process (**A**), cellular component (**B**), molecular function (**C**) gene ontology (GO) terms are shown in bubble plots, with Venn diagrams depicting the specific overlap of these GO terms that were associated with the Susceptible (blue) or Intermediate (violet) subgroups compared to Resistant. (**A**) Biological processes mostly overlap between two subgroup comparisons (14/15), as do (**B**) the cellular components terms (12/15) and (**C**) molecular processes (12/15). The interesting differences between downregulated genes within these comparisons include (**A**) aerobic electron transport chain, (**B**) organelle envelope and (intracellular) membrane-bound organelle, (**C**) 2 iron 2 sulfur cluster binding, electron transfer activity and enzyme binding downregulated in Susceptible; and (**A**) ribonucleoprotein complex biogenesis, (**B**) respiratory chain complex, NADH dehydrogenase complex and oxidoreductase complex, (**C**) proton channel activity, ubiquitin-protein transferase inhibitor and regulator activity downregulated in Intermediate (see bold text).

**Reference for Supplementary Figure 1:**

Huang, K., Milton, L. K., Dempsey, H., Power, S. J., Conn, K. A., Andrews, Z. B., & Foldi, C. J. (2023). Rapid, automated, and experimenter-free touchscreen testing reveals reciprocal interactions between cognitive flexibility and activity-based anorexia in female rats. *Elife*, *12*. <https://doi.org/10.7554/eLife.84961>
